# Eradicating cancer cells: struggle with a chameleon

**DOI:** 10.18632/oncotarget.222

**Published:** 2011-02-28

**Authors:** Jiabo Di, Tjitske Duiveman-de Boer, Carl G. Figdor, Ruurd Torensma

**Affiliations:** Department of Tumorimmunology, Radboud University Nijmegen Medical Centre, Nijmegen, The Netherlands

## Abstract

Eradication of cancer stem cells to abrogate tumor growth is a new treatment modality. However, like normal cells cancer cells show plasticity. Differentiated tumor stem cells can acquire stem cell properties when they gain access to the stem cell niche. This indicates that eradicating of stem cells (emptying of the niche) alone will not lead to eradication of the tumor. Treatment should be directed to cancer stem cells ànd more mature cancer cells.

The discovery of cancer stem cells for solid tissues evoked an enormous research effort into this new avenue of treatment options [1]. Especially, the properties of cancer stem cells gained an enormous welcome because several in vivo findings could be explained. Cancer stem cells are resistant to irradiation and survive chemotherapeutic agents due to mechanisms very well known from normal stem cells. This in contrast to their mature offspring. Those stem cell features explain that- in a high percentage of patients- after killing the more mature tumor cells with these treatment modalities, the tumor will regrow. The first compounds that show specific killing of cancer stem cells are reported [2]. Such experiments suggest that the cancer stem cells could be killed given the right drugs are used [3]. Salinomycin one of the reported cancer stem cell drugs make stem cells that express multidrug transporters again vulnerable for chemotherapeutic drugs by blocking the drug expelling ABC-transporter [4]. Expression of the ABCB5 transporter was reported to be confined to melanoma stem cells [5] and used as a target to eradicate cancer stem cells[6]. Also ALDH positive cells were shown to be enriched in tumor initiating cells[7].

However, for melanoma the cancer stem cell concept is challenged. Initially it was demonstrated using NOD/SCID mice that one in approximately one million cancer cells was able to evoke a tumor in those mice [8-10]. This frequency was challenged when other recipient mice were used and the tumor cells were implanted in matrigel [11-13]. This got recently a follow up with CD271 positive melanoma cells were the tumor initiating cells as deduced from an impressive number of different cancer cell lines cultured in vitro but also from cancer cells directly obtained from freshly excised tumors [14]. The ABCB5 positive fraction could be further enriched when the expresion of the VEGFR was taken into account [15]. However, this was challenged by other researchers [16].

One reason that increases tumor initiating cell frequency is the immune status of the mouse used for those experiments. Initially NOD/SCID mice that lack B- and T-lymphocytes were used. Later on more highly immunocompromised NOD/SCID interleukin-2 receptor gamma chain null (Il2rγ(-/-) mice, which also lack NK cells were used. Such studies clearly demonstrate that heterogeneity exists in tumors: a population of cells that initiates tumors due to lack of immune surveillance whereas a less abundant population resists a better equipped immune system. Another reason for this difference in frequency of cancer initiating cells could rely in the plasticity of stem cells. The normal route for a stem cell is to differentiate from stem cell to mature tissue cells and is paved with several proliferation and maturation/differentiation steps. Several points in this differentiation are believed to be unidirectional, once taken no return is possible (lineage-commitment) [17]. Observed transdifferentiation was shown to be due to fusion of implanted stem cells with the diseased muscle or liver cells [18-20]. There are, however, data out that this is not as strict as propagated. Hematopoietic stem cells were able to dedifferentiate and become liver cells [21]. Knocking down JARID1B in slow cycling melanoma cells exhausted the tumor However, expression of JARID1B is dynamic since negative cells can become JARID1B positive [10]. Fibroblasts could transdifferentiate into cardiomyocytes [22]. Fibroblasts were even able to become blood cells without reprogramming into an iPS cell first [23] and endothelial cells could simply be converted into multipotent stem-like cells by transforming growth factor β2 or bone morphogenetic protein 4 [24]. Also in the spermatogonial development more differentiated cells can go back to the stem cell state when the stem cell niche is emptied and the number of stem cells is decreased. Moreover, transient amplyfying cells in the gut require again stem cell properties when they contact paneth cells that supply them with Wnt and rescue the stem cell status. In this way the normal number of stem cells is recovered by differentiated stem cells that regain stem cell properties [25]. For melanoma such a mechanism could also be applicable. Dedifferentiation of more differentiated cells will level a shortage of stem cells. Just like a chameleon changes its color depending on the circumstances. If present, such plasticity would have major implications for therapeutic approaches that target only cancer stem cells. A combination therapy of destroying cancer stem cells as well as more mature progeny could wipe out the source of cancer cells. Moreover, as indicated above that more immune mice reject more tumorinitiating cells, enforcement of the immune system should aid in better survival of patients [26].
